# Oxidative stress drives mutagenesis through transcription-coupled repair in bacteria

**DOI:** 10.1073/pnas.2300761120

**Published:** 2023-06-26

**Authors:** Juan Carvajal-Garcia, Ariana N. Samadpour, Angel J. Hernandez Viera, Houra Merrikh

**Affiliations:** ^a^Department of Biochemistry, Vanderbilt University School of Medicine, Nashville, TN 37232; ^b^Department of Microbiology, University of Washington, Seattle, WA 98195

**Keywords:** mutagenesis, evolution, antibiotic resistance, transcription coupled repair

## Abstract

In bacteria, mutations lead to the evolution of antibiotic resistance, which is one of the main public health problems of the twenty-first century. Therefore, determining which cellular processes most frequently contribute to mutagenesis, especially in cells that have not been exposed to exogenous DNA damage, is critical. Here, we show that endogenous oxidative stress is a key driver of mutagenesis and the subsequent development of antibiotic resistance. This is the case for all classes of antibiotics and highly divergent species tested, including patient-derived strains. We show that the transcription-coupled repair pathway, which uses the nucleotide excision repair proteins (TC-NER), is responsible for endogenous oxidative stress-dependent mutagenesis and subsequent evolution. This suggests that a majority of mutations arise through transcription-associated processes rather than the replication fork. In addition to determining that the NER proteins play a critical role in mutagenesis and evolution, we also identify the DNA polymerases responsible for this process. Our data strongly suggest that cooperation between three different mutagenic DNA polymerases, likely at the last step of TC-NER, is responsible for mutagenesis and evolution. Overall, our work identifies a highly conserved pathway that drives mutagenesis due to endogenous oxidative stress, which has broad implications for all diseases of evolution, including antibiotic resistance development.

## Significance

A common way of becoming resistant to antibiotics is mutations in the bacterial genome. However, how these mutations arise remains poorly understood. Here, we determine that endogenous oxidative stress is the main driver of mutagenesis that leads to antibiotic resistance development. In addition, we find that a highly conserved DNA repair pathway, transcription-coupled repair, is responsible for this oxidative stress-driven evolution of antibiotic resistance. Our prior work showed that the Mfd protein, which contributes to TC-NER, accelerates the development of antibiotic resistance. The work presented here shows that, in fact, all of the proteins involved in TC-NER are contributing significantly to resistance development, expanding our knowledge of the fundamental mechanisms driving not only drug resistance, but also evolution in general.

Mutations provide the necessary genetic diversity that natural selection can then use to help organisms adapt to new environments. Even though mutations are mostly deleterious, and lower mutation rates are generally accepted to be beneficial, all organisms have a baseline mutation rate that allows them to evolve (1). Multiple processes are known to contribute to spontaneous mutagenesis: transcription, replication errors, or spontaneous damage to the DNA that leads to erroneous repair (2, 3). However, which mechanisms most commonly lead to mutations and drive evolution remain unknown (3).

An important source of mutations is damage to the DNA. Some forms of DNA damage are cytosine deamination, depurination, or single-stranded breaks (which can then be converted into double-stranded breaks) (4, 5). However, which of these types of damage cells are most commonly exposed to is unclear. It is important to determine this because these processes will ultimately lead to mutagenesis. Our lack of knowledge regarding the frequency of which types of DNA damage are most commonly faced by cells is partially due to the fact that most studies investigating the mechanisms of mutagenesis in bacteria have been performed upon exposing cells to high amounts of exogenous DNA damage, such as UV light. However, to better understand the mechanisms of mutagenesis, which will ultimately drive evolution, we need to perform these studies in cells that are not exposed to exogenous DNA damage.

Oxidative stress is considered to be one of the main sources of endogenous DNA damage in bacteria (6). Oxidative stress is an obligatory consequence of aerobic respiration and metabolic processes, and it results from an imbalance between highly reactive oxidative molecules (such as reactive oxygen species, ROS) and the cell’s ability to detoxify them (7). These oxidative molecules can react with biomolecules like proteins, lipids, and DNA, changing their chemical structure and damaging them. In the case of DNA, if this damage does not get properly repaired, oxidative stress could be a significant driver of mutations.

Accordingly, bacterial cells lacking catalases and superoxide dismutases, enzymes that de-toxify ROS, show growth defects as well as increased mutagenesis, even in the absence of exogenous DNA damage (8–10). Moreover, cells lacking MutM and MutY, glycosylases involved in the repair of oxidative DNA damage, show increased mutation rates (11, 12). We therefore decided to test the role that endogenous oxidative stress plays in bacterial evolution.

To test our model, we utilized antibiotics and found that oxidative stress is indeed a major source of spontaneous mutations that ultimately drive evolution in aerobic bacterial cultures. We determined that the observed oxidative stress-dependent evolution is driven by nucleotide excision repair (NER). This is despite NER being involved in the removal and repair of bulky or helix-distorting DNA damage and not oxidative DNA damage, which is commonly repaired by base excision repair (BER) (13, 14). Surprisingly, we observed that NER has a detrimental effect on bacterial cells, as it promotes mutagenesis that is caused by endogenous oxidative stress. We found that mutations arising from NER depend on cooperation between three different polymerases, including Y-family DNA polymerases. NER is a highly conserved DNA repair pathway that recognizes and excises DNA damage. The damage recognition step is carried out by UvrA either globally (directly on DNA) or upon interaction with a stalled RNA polymerase (transcription-coupled repair, TCR). Critically, we found that the driving force behind NER-driven mutagenesis and evolution is strongly tied to transcription, highlighting TCR as the major contributor to oxidative-stress–driven evolution.

## Results

### Oxidative Stress Drives the Evolution of Antibiotic Resistance.

Oxidative stress has been proposed to be an important source of endogenous DNA damage in bacteria (7). For this reason, we considered whether decreasing the amount of oxidative stress bacterial cells are exposed to would have an effect on the kinetics of evolution. We utilized a previously described laboratory evolution assay to test this hypothesis (15). During this assay, we measured adaptation to the transcription inhibitor rifampicin in four different, highly divergent species: *Bacillus subtilis*, a multidrug-resistant strain of *Staphylococcus aureus*, *Salmonella enterica* serovar Typhimurium, and *Pseudomonas aeruginosa*. We have previously shown that the increase in the minimal inhibitory concentration (MIC) observed over time correlates with the appearance of mutations in known resistance genes (15, 16).

Given that ROS has been predicted to be a major driver of DNA damage, we chose to test our hypothesis using rifampicin. Unlike some other antibiotics, rifampicin does not increase the amount of ROS in cells (17), and therefore, using it for our initial experiments assured that any impact of oxidative stress we observe on evolution would most likely have an endogenous origin.

To test the impact of ROS on evolution, we performed our experiments using cells that were not treated with any antioxidants, and in parallel, we decreased the amount of oxidative stress by adding the antioxidant thiourea, which has been used in the past to reduce oxidative stress in bacteria (17–19) a concentration of thiourea that does not affect the growth rate (*SI Appendix*, Fig. S1 *A*–*D*) or the MIC_50_ of rifampicin (*SI Appendix*, Fig. S1 *E*–*H*). In addition, to avoid potential artifacts that thiourea may impose on cells, in parallel, we also overexpressed the *katA* gene, which encodes for the ROS scavenging protein catalase, in *B. subtilis* (*SI Appendix*, Fig. S1 *A*, *E*, *I*, and *J*) (20). Last, for the two facultative anaerobic species, *S. aureus* and *S. enterica*, we also performed the experiment in anaerobic conditions. As a control, we also confirmed that sublethal amounts of rifampicin don’t increase ROS in *S. enterica* (*SI Appendix*, Fig. S1*L*).

**Fig. 1.**
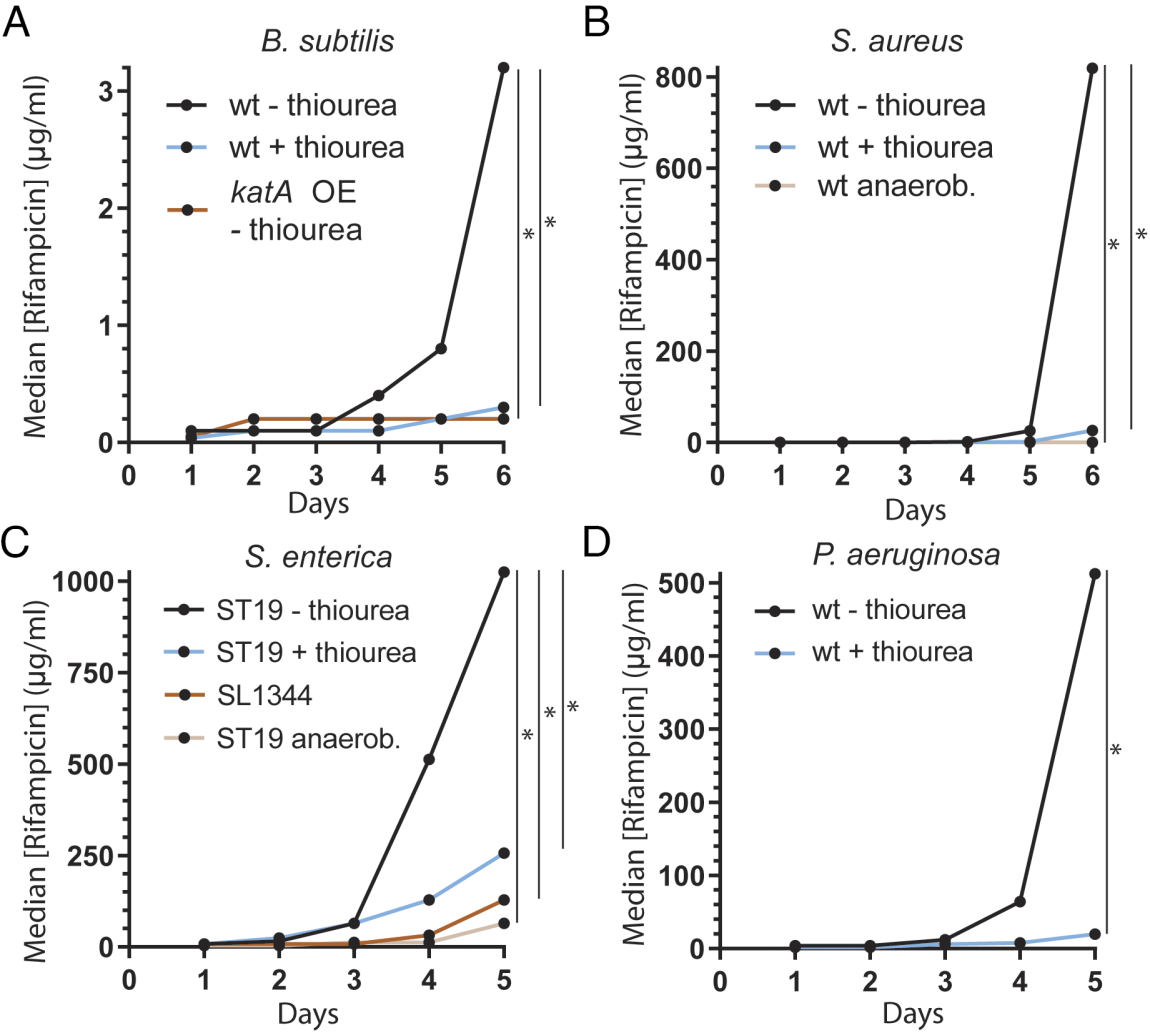
Oxidative stress drives the evolution of antibiotic resistance. Median concentration of rifampicin that allows for growth in the indicated strains at each sampled timepoint. 50 mM (*A*–*C*) or 10 mM (*D*) thiourea was included in the media when indicated. 1 mM IPTG was added for *katA* overexpression. n = 23 (*B. subtilis* – thiourea, rifampicin), 12 (*B. subtilis* + thiourea, rifampicin), 24 (*B. subtilis katA* overexpression, rifampicin), 12 (*S. aureus* – thiourea), 12 (*S. aureus* + thiourea), 12 (*S. aureus* anaerobiosis), 35 (*S. enterica* serovar Typhimurium ST19), 34 (*S. enterica* serovar Typhimurium ST19 + thiourea), 24 (*S. enterica* serovar Typhimurium *SL1344*), 12 (*S. enterica* serovar Typhimurium ST19), 22 (*P. aeruginosa* – thiourea), 12 (*P. aeruginosa* + thiourea) biological replicates. Statistical significance was assessed with a two-tailed Mann–Whitney *U* test, *P < 0.05.

The starting antibiotic concentration in which the four bacterial species were able to survive was 0.025 µg/mL (*B. subtilis*), 3.125 ng/mL (*S. aureus*), 4 µg/mL (*S. enterica*), and 4 µg/mL (*P. aeruginosa*) of rifampicin (Fig. 1 and *SI Appendix*, Fig. S1 *E*–*H*). After 35 to 40 generations in culture, the median concentrations of antibiotic-treated cells were able to withstand increased to 3.2 µg/mL, 820 µg/mL, 1,024 µg/mL, and 512 µg/mL of rifampicin respectively (Fig. 1). This value was significantly lower in cells that had been exposed to thiourea: 0.3 µg/mL (*B. subtilis*), 26.4 µg/mL (*S. aureus*), 256 µg/mL (*S. enterica*), and 20 µg/mL (*P. aeruginosa*) of rifampicin. The overexpression of *katA* in *B. subtilis*, as well as performing the assay in anaerobiosis in *S. aureus* and *S. enterica*, had a similar effect, as the median MIC on the last day of the experiments was 0.2 µg/mL, 0.4 µg/mL, and 64 µg/mL of rifampicin respectively (Fig. 1*A*).

Interestingly, inactivating mutations in another gene (*katE*) that codes for a catalase have been found in patient-derived *S. enterica* serovar Typhimurium strains (21), and this is the case with the strain that we used (ST19) (22). When we performed the same experiment in a strain with a functional KatE protein (SL1344) (22), which we confirmed has a higher catalase activity (*SI Appendix*, Fig. S1*K*), we observed a reduction in the kinetics of evolution, similar to the addition of thiourea (Fig. 1*C*). These observations are consistent with endogenous oxidative stress driving evolution generally in bacteria.

To test whether this phenomenon is universally conserved and not unique to the transcription inhibitor rifampicin, and because transcription-coupled repair has been shown to promote the evolution of antibiotic resistance (15), we performed the same evolution assays using three other classes of antibiotics. We observed similar results when we used the translation inhibitor kanamycin and the folate synthesis inhibitor trimethoprim in *B. subtilis,* as well as the cell wall synthesis inhibitor phosphomycin in *S. aureus* (*SI Appendix*, Fig. S2 *A*–*H*). Altogether, our results suggest that ROS plays a critical role in evolution across highly divergent bacteria.

### TCR Drives Oxidative Stress-Dependent Evolution.

We next decided to determine the mechanism by which endogenous oxidative stress drives evolution, using the genetically tractable species *B. subtilis*. Because oxidative DNA damage is commonly repaired by BER, we first tested whether BER mutants have decreased mutation rates, which would correlate with slower evolution of resistance. However, and consistent with previous reports (23, 24), we observed that strains lacking the DNA glycosylases MutY and MutM have higher mutation rates than wild-type cells (*SI Appendix*, Fig. S3*A*), suggesting BER is not the source of ROS-induced mutagenesis and evolution.

We and others have previously shown that, in the absence of exogenous DNA damage, the bacterial TCR protein Mfd promotes mutagenesis across many different bacterial species (15, 25, 26). In addition, we previously showed that this pro-mutagenic effect depends on the interaction of Mfd with the RNA polymerase (RNAP) and the NER protein UvrA (15). Therefore, we decided to focus on NER. This DNA repair pathway has been shown to cause spontaneous mutagenesis in some bacteria (27–29), even though it has a protective effect against mutations when bacteria are exposed to DNA-damaging agents (30, 31).

Bacterial NER has traditionally been described as consisting of two subpathways, global genome repair (GGR), whereas transcription-coupled repair (TCR), differing in the damage recognition step (13). In GGR, UvrA scans the genome and binds DNA to trigger NER, and in TCR it is a stalled RNA polymerase that either through UvrD or the protein Mfd recruits the NER machinery to the site of DNA damage (32). This model has been put into question by recent studies claiming that, in bacteria, most NER is coupled to transcription and that if any, GGR has a minor role in this process (33, 34).

**Fig. 2.**
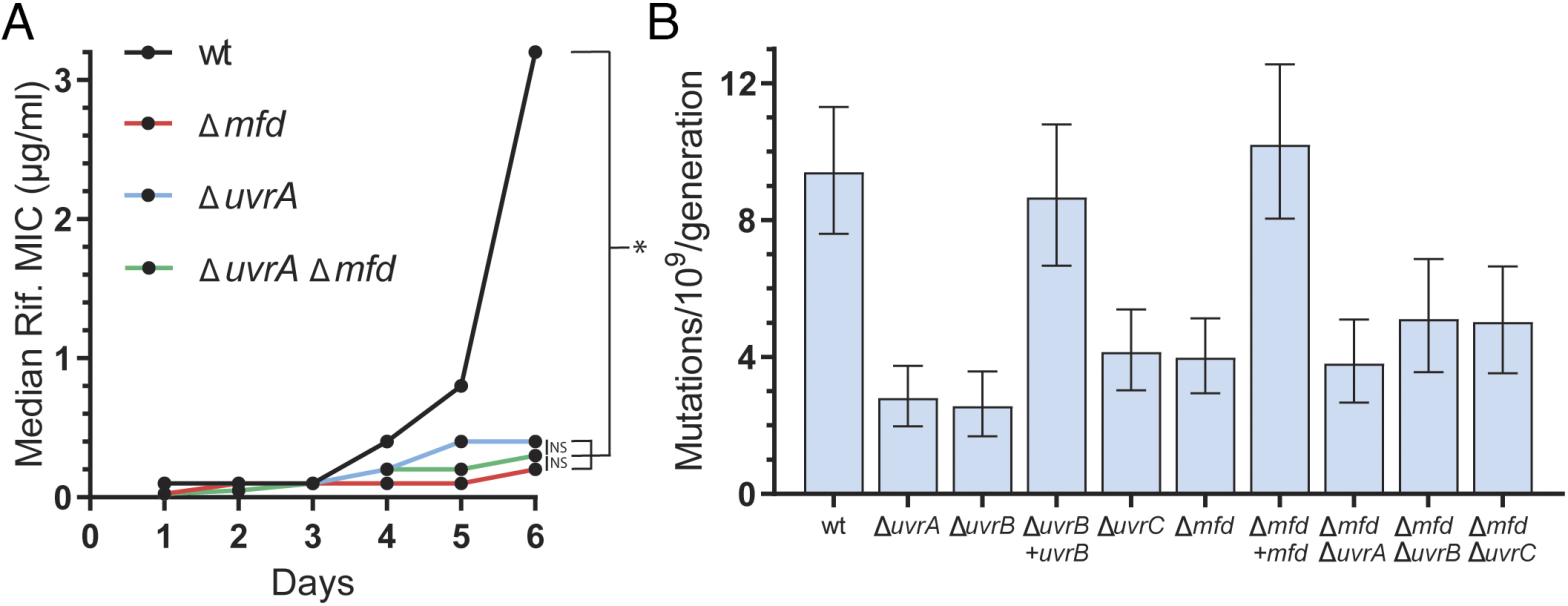
Transcription-coupled repair promotes mutagenesis. (*A*) Median rifampicin concentration that allows for growth in the indicated strains at the indicated timepoints. n = 23 (wt), 36 (Δ*uvrA*), 24 (Δ*mfd*), 12 (Δ*uvrA* Δ*mfd*) biological replicates. (*B*) Mutation rates of *B. subtilis* strains measured using rifampicin. n = 54 (wt), 48 (Δ*uvrA*), 37 (Δ*uvrB*), 47 (Δ*uvrB* + *uvrB),* 48 (Δ*uvrC*), 59 (Δ*mfd*), 49 (Δ*mfd* + *mfd*), 40 (Δ*mfd* Δ*uvrA*), 40 (Δ*mfd* Δ*uvrB*), 50 (Δ*mfd* Δ*uvrC*) biological replicates. Statistical significance was assessed with a two-tailed Mann–Whitney *U* test, *P < 0.05. Error bars are 95% CI.

We performed an evolution assay in wild-type *B. subtilis* cells and in isogenic strains lacking the core component of the NER machinery UvrA, and we observed that UvrA promotes the evolution of antibiotic resistance (Fig. 2*A*). In addition, we measured mutation rates using the Luria–Delbruck fluctuation assay (35) in wild-type and mutants for the core NER proteins UvrA, UvrB, and UvrC in the absence of exogenous DNA damage. We observed a 50 to 75% decrease in the mutation rates in NER-deficient strains (Fig. 2*B*), indicating that NER promotes spontaneous mutagenesis in *B. subtilis*.

To test whether TCR was solely responsible for NER-dependent mutagenesis, we built double mutants that lacked Mfd, and the Uvr proteins. If NER is mutagenic only due to TCR, then we expected that the double mutants lacking Mfd and NER proteins would have an epistatic relationship, and that the effect of the double mutants in mutagenesis and evolution would be similar to the single mutants. On the other hand, if NER-mediated mutagenesis is through both GG-NER and TCR, the combination of mutants lacking both Mfd and NER proteins would have a further reduced mutation rates. To discern between these possibilities, we performed evolution and mutation rate assays in *B. subtilis* cells lacking Mfd, as well as UvrA and Mfd both and observed a comparable decrease in the evolution of resistance in both single mutants and in the double mutant (Fig. 2*A*). Consistently, the mutation rates of the single and double mutants side-by-side did not decrease further. We found that the mutation rates of strains lacking both Mfd and any one of the three canonical NER factors have the same mutation rates as each single mutant alone (Fig. 2*B*). This strongly suggests that all (mutagenic) NER is coupled to transcription.

Additionally, we measured mutation rates in *S. enterica* serovar Typhimurium cells lacking either UvrB or Mfd and compared them to isogenic wild-type cells. We observed a similar result as in *B. subtilis*: in the absence of either UvrB or Mfd there was a similar decrease in mutation rates compared to a wildtype strain. These results suggest that the mutagenicity of TC-NER is conserved in the highly divergent bacteria *B. subtilis* and *S. enterica* (*SI Appendix*, Fig. S3*B*).

**Fig. 3.**
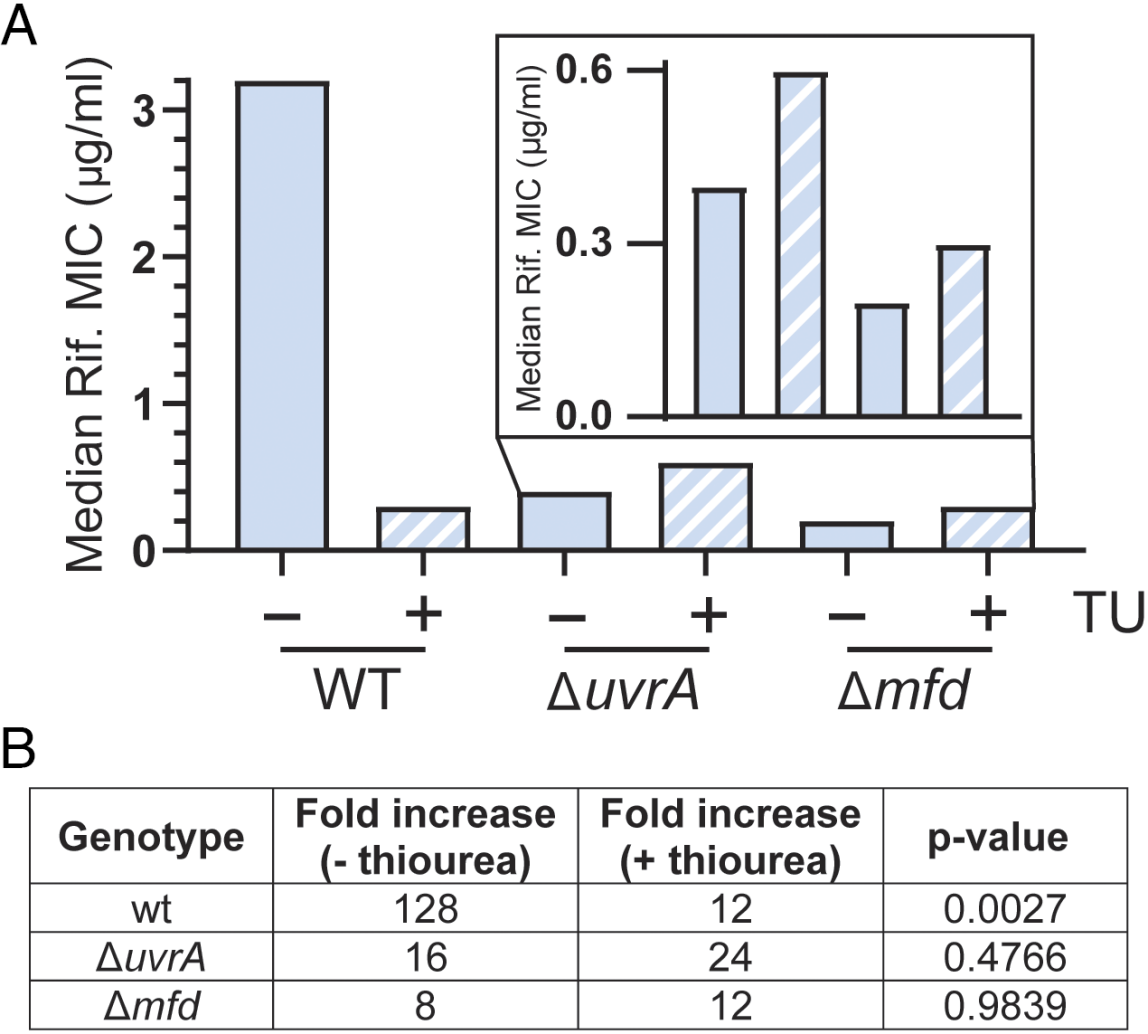
TCR promotes oxidative stress-dependent mutagenesis. (*A*) Median rifampicin concentration that allows for growth in the indicated strains after 6 d of evolution. 50 mM thiourea was included in the media when indicated. n = 23 (wt − thiourea), 12 (wt + thiourea), 34 (Δ*uvrA* − thiourea), 12 (Δ*uvrA* + thiourea), 24 (Δ*mfd* − thiourea), 12 (Δ*mfd* + thiourea) biological replicates. (*B*) Fold increase in the median rifampicin concentration that allows for growth in the indicated strains after 6 d of evolution in the presence or absence of 50 mM thiourea. Statistical significance was assessed with a two-tailed Mann–Whitney *U* test.

For both treatment with thiourea or *katA* overexpression, the magnitude of the decrease in the rate of evolution was similar to the one observed in strains lacking UvrA and/or Mfd (Figs. 1*A* and 2*A*). We therefore tested whether TCR is responsible for the mutagenic effect of endogenous oxidative damage by performing evolution assays in strains deficient in TCR genes (Δ*uvrA* and Δ*mfd*) in the presence or absence of thiourea. Although these strains have a serious deficiency in evolving resistance to antibiotics, toward the end of the evolution assays, a slight increase in their MIC can be observed (Figs. 2*A* and 3). We took advantage of this and analyzed the rate at which evolution starts to take off at the last time points when oxidative stress is reduced. Consistent with our model, we observed that, in strains lacking UvrA or Mfd, thiourea did not have any effect on the rate of evolution (Fig. 3). This strongly suggests that endogenous oxidative stress is driving mutagenesis and subsequent evolution almost exclusively through TC-NER.

### A Replicative and Two Y-Family Polymerases Function in the Same Pathway as NER.

**Fig. 4.**
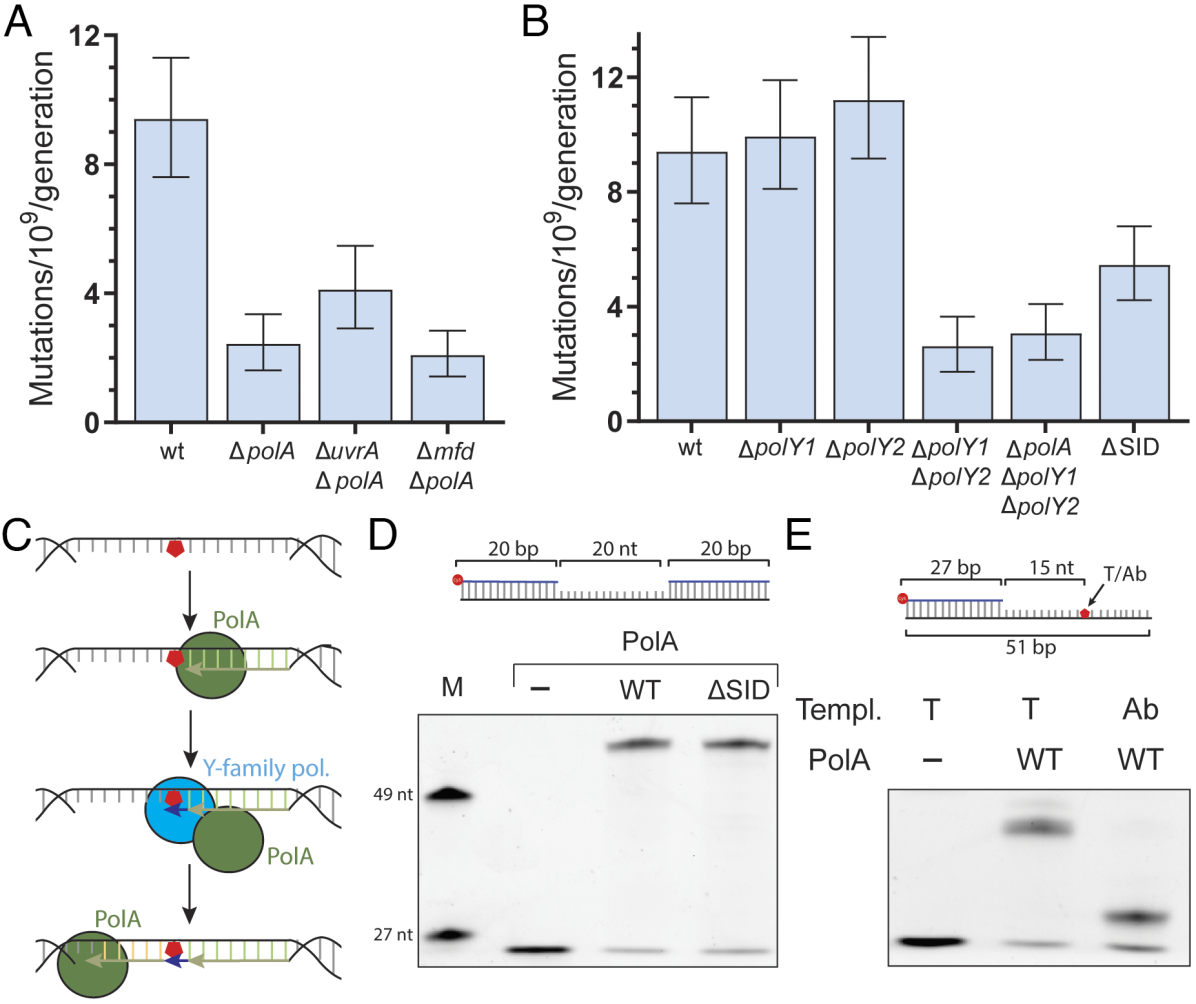
Three polymerases are required for TCR mutagenesis. (*A* and *B*) Mutation rates of *B. subtilis* strains. N = 54 (wt), 40 (Δ*polA*), 36 (Δ*uvrA* Δ*polA*), 44 (Δ*mfd* Δ*polA*), 57 (Δ*polY1*), 56 (Δ*polY2*), 35 (Δ*polY1* Δ*polY2*), 43 (Δ*polA* Δ*polY1* Δ*polY2*) biological replicates. Error bars are 95% CI. (*C*) Model for the molecular mechanism of NER-dependent mutagenesis. (*D*) Primer extension assay with purified *B. subtilis* PolA (middle lane) or PolA-ΔSID (right lane) using a gap substrate. Due to DNA being single stranded in the transcription bubble and/or during NER, the nontranscribed strand is prone to damage that stalls PolA and leads to the recruitment of Y-family polymerases, further increasing the possibility of acquiring a mutation. (*E*) Primer extension assay with purified *B. subtilis* PolA using a substrate that has either a thymidine (middle lane) or an abasic site (right lane) in its ninth nucleotide.

We set out to determine the molecular mechanism behind NER-dependent mutagenesis. During NER, UvrA and UvrB recognize the lesion, recruit UvrC which then excises the part of the DNA containing the lesion. This leaves a gap that then needs to be filled in by a DNA polymerase. We reasoned that this gap-filling step of NER is the most likely source of mutations and that it is likely due to the activity of an error-prone DNA polymerase. Based on in vitro experiments, classically, DNA polymerase I (PolA in *B. subtilis*) is thought to perform this step (36, 37). Thus, we measured mutation rates in cells lacking PolA. Unlike *E. coli*, *B. subtilis* cells lacking PolA are viable and display no growth defects. This is thought to be because the 5′→3′ Okazaki fragment maturation activity of PolA can be performed by the ExoR exonuclease (38–40). Although in vitro work had led to the conclusion that PolA is a high-fidelity polymerase (41), we found that this is not the case in vivo. We observed that PolA promotes mutagenesis, as cells lacking PolA showed a decrease in mutation rates that were very similar to that seen in NER-deficient strains (Fig. 4*A*).

To determine whether NER is mutagenic due to PolA activity, we measured the mutation rates of *uvrA polA* and *mfd polA* double knockouts. When we compared the mutation rates of strains lacking either PolA, Mfd, or UvrA alone to the double mutants that lacked Mfd and PolA, as well as UvrA and PolA, we did not see an additional decrease in mutation rates, indicating that Mfd-associated, mutagenic NER is in the same pathway as PolA (Fig. 4*A*).

However, in vitro studies with the *E. coli* homolog of PolA (PolI) have has determined to be high-fidelity, making it unlikely that by itself, it would introduce an error in such a small gap as the one generated during NER (41). Given that previous work has suggested that *B. subtilis* PolA interacts with two error-prone, Y-family polymerases, PolY1 and PolY2 (orthologs of the *E. coli* PolIV and PolV and the mammalian Pol kappa and Pol eta) (38), we reasoned that these Y-family polymerases could also be involved in the pro-mutagenic nature of NER. To test our model, we generated strains that lacked either PolY1, PolY2, or both polymerases. When we determined the mutation rates of strains that either lacked PolY1 or PolY2, we did not observe a decrease in mutation rates in either single mutant. Interestingly we did observe a decrease in mutation rates in strains lacking both PolY1 and PolY2, suggesting a redundant, promutagenic role for these polymerases (Fig. 4*B*). However, whether they function in TC-NER remained unclear.

If our hypothesis that these polymerases cooperate with PolA during the NER gap filling step is correct, then in strains that lack all three polymerases, we should not observe any additional decrease in mutation rates. Indeed, we observed that there was no additional decrease in mutation rates when cells lacked all three polymerases compared to cells either lacking only PolA, the Uvr proteins, or both PolY1 PolY2 (Fig. 4*B* and *SI Appendix*, Fig. S3*D*). Therefore, we conclude that these polymerases are in the same pathway and cooperate to complete the last step during NER. The observed requirement for both an A-family replicative polymerase and a Y-family polymerase led us to the model outlined in Fig. 4*C*, in which PolA performs DNA synthesis during NER but will stall if a DNA lesion is present on the nontranscribed, NER template strand. This stalled PolA would then recruit a Y-family polymerase to overcome the lesion, increasing the chances of generating a mutation. The secondary lesion on the nontranscribed strand could occur when this region of the genome is single stranded during transcription or NER, which would render it more susceptible to damage (42). To test this model, we developed a biochemical assay with purified *B. subtilis* PolA (*SI Appendix*, Fig. S3*C*). Using an in vitro primer extension assay on a ssDNA gap template similar to the one that would be generated during NER we examined whether *B. subtilis* PolA can fill in this gap. We indeed observed that *B. subtilis* PolA is able to efficiently fill in this gap, as well as displace the complementary strand downstream of the ssDNA gap, a known property of the *E. coli* homolog (43) (Fig. 4*D*).

To then test whether a common ROS lesion can stall PolA, we measured DNA synthesis on an ssDNA template containing an abasic site, one of the most common lesions observed in DNA (44), and a substrate for PolV in *E. coli* (45). We observed that this form of DNA damage is a strong block to synthesis by PolA (Fig. 4*E*), supporting the model that PolA alone cannot fill in a gap generated during NER if there is damage to the nontranscribed strand.

Last, we created a 10 amino acid deletion in the endogenous *polA* gene that includes the predicted region of interaction between PolA and PolY1/2 (specific interaction domain, SID) (38), and observed that it leads to a decrease in mutation rates compared to wild-type cells (Fig. 4*B*). However, this decrease is smaller than a full *polA* deletion, which suggests that we are not destroying the interaction completely. Critically, we purified the PolA-ΔSID mutant and observed no difference with the wildtype protein in its ability to synthesize DNA using the ssDNA gap substrate (Fig. 4*D* and *SI Appendix*, Fig. S3*C*), indicating that this decrease is not due to loss of PolA synthesis ability. This further supports our model for the involvement of both the high-fidelity PolA and the low-fidelity Y-family polymerases in the gap-filling step of NER.

## Discussion

Mutations generate the genetic diversity that evolution requires. Damage to the DNA is an important source of mutations, and since most organisms are not exposed to exogenous DNA damage, endogenous sources of damage likely play a key role in evolution. In this work, we identified oxidative stress as the main source of endogenous DNA damage leading to evolution in bacteria. Reactive oxygen species have been shown to lead to the most spontaneous mutagenesis in *E. coli* (46) and are thought to be an important source of endogenous DNA damage (7). We tested the contribution of oxidative stress to mutagenesis by measuring the evolution of resistance to antibiotics in cells that have reduced oxidative stress, by either growing them in the presence of thiourea or overexpressing the catalase gene *katA.* We observed that the evolution of antibiotic resistance was slower in these conditions in diverse bacteria, including patient-derived strains (Fig. 1). Sublethal concentrations of certain antibiotics have been shown to lead to oxidative stress, and this phenomenon has been proposed to lead to antibiotic resistance (17, 18). However, we observe that oxidative stress leads to the evolution of resistance to an antibiotic that does not seem to increase the production of reactive oxygen species (ROS), such as rifampicin (*SI Appendix*, Fig. S1*L*) (17).

We then show that nucleotide excision repair (NER), which strongly suppresses mutagenesis in cells exposed to DNA-damaging agents (30, 31), is actually promoting mutagenesis in the absence of exogenous mutagens and is generally a promutagenic mechanism. Bacteria lacking any one of the three core components of the NER mechanism, UvrABC, have lower mutation rates than wildtype cells, indicating that NER causes spontaneous mutations (Fig. 2). In addition, our data indicate that in the absence of exogenous damage, all mutagenic NER functions in the same pathway as the transcription-coupled (TC) NER factor Mfd, suggesting that mutagenic NER is universally transcription dependent (Fig. 2). This is consistent with recent biochemical findings which suggest most bacterial NER is transcription dependent (33). Interestingly, the little evolution we observe in NER-deficient strains is *not* diminished in the presence of thiourea (Fig. 3), suggesting that oxidative stress-driven evolution is dependent on NER.

Our data shows that it is the cooperation of at least two DNA polymerases that causes NER-dependent mutations: the replicative polymerase commonly associated with NER, PolA, and one of two redundant Y-family polymerases, PolY1 and PolY2 (Fig. 4). We propose that DNA damage in the NER template strand (the noncoding strand) explains this requirement for both DNA polymerases to complete NER (Fig. 4*D*), which will naturally lead to an increased likelihood of mutations being introduced into the synthesized DNA gap. A similar model has been proposed to explain observations of mutagenesis in prokaryotes and eukaryotes, but in the presence of exogenous DNA damage (47, 48), including in a recent preprint that uses a mouse liver cancer model in which cells are exposed to high levels of the DNA damaging agent diethylnitrosamine (49). This suggests that this mechanism of mutagenesis is universally conserved.

In our model, the DNA lesion that is encountered by PolA, requires the action of TLS polymerases, and leads to mutagenesis, is independent of the lesion that triggered NER. We propose that this second lesion may be caused during transcription and/or the excision of a damaged oligonucleotide by NER. The nontranscribed strand stays as ssDNA for an extended period of time during both processes, and it is well-known that ssDNA is more prone to oxidative damage than dsDNA (50, 51). Alternatively, the NER machinery has been shown to excise nondamaged DNA in vitro (52) and transcription stimulates this process in vivo (53). These gratuitous repair events, even if much less efficient than the excision of damaged DNA, are predicted to be a common phenomenon, as the amount of nondamaged DNA outweighs the amount of damaged DNA by several orders of magnitude in cells that are not exposed to exogenous DNA damaging agents (54). Moreover, Mfd has been found to be bound to DNA throughout the genome in the absence of exogenous DNA damage (55, 56), and it plays a role in transcription that is independent of its role in TC-NER (57). This constitutive association with DNA and RNAP could lead to excision and fill in synthesis, therefore increasing the likelihood of mutations being introduced without the need for a DNA lesion present in close proximity and on the opposite strand. Another possibility is that, as ROS can damage the deoxynucleotide pools, PolA and/or the Y-family polymerases could be using an oxidized dNTP to fill the gap, which could then lead to mutagenesis during the next round of replication (58).

Last, we have described in a recent preprint a small molecule inhibitor of Mfd, which delays the evolution of antibiotic resistance in many different pathogenic bacterial species (16). By identifying other factors in the Mfd-dependent, promutagenic pathway, we have expanded the potential targets for an antievolution drug that can be used to minimize antibiotic resistance generation during the treatment of infections in the clinic (16).

## Materials and Methods

### Bacterial Culture.

*B. subtilis*, *S. enterica* serovar Typhimurium, and *S. aureus* were cultured in lysogeny broth (LB), and *P. aeruginosa* in LB with 0.1% Tween 20 (when liquid media). Bacterial plates were grown overnight at 37 °C unless otherwise indicated with the following antibiotics when appropriate: 500 µg/mL erythromycin and 12.5 mg/mL lincomycin (MLS), 5 µg/mL kanamycin (*B. subtilis*) or 50 µg/mL (*E. coli* and *S. enterica*) kanamycin, 25 µg/mL chloramphenicol, and 100 µg/mL carbenicillin. When grown in liquid media, cultures were started from single colonies and were grown with aeration (260 rpm). A list of all strains used in this study can be found in *SI Appendix*, Table S1.

### Strain Construction.

The parental strain for all *B. subtilis* strains used in this study is HM1 (same as AG174, originally named JH642) (59, 60). Gene deletions that are marked with MLS or kanamycin resistance were obtained from ref. 61. Genomic DNA from these strains was purified with the GeneJET Genomic DNA Purification Kit (Thermo) following the manufacturer’s instructions and transformed into the HM1 background as in previously described (62). When necessary to make strains that carry multiple mutations, these antibiotic-resistant cassettes were excised by transforming the strains with a plasmid expressing the Cre recombinase (pDR244, BGSCID: ECE274) purified from RecA+ *Escherichia coli* (NEB) cells with the GeneJET Plasmid Miniprep Kit (Thermo), generating markerless strains (61). Recombinants containing markerless deletions were checked by PCR (*SI Appendix*, Table S2).

For *katA* overexpression and complementation experiments, the coding sequence of appropriate the gene was amplified using Q5 polymerase (NEB) (*SI Appendix*, Table S2) and cloned between the *Hind*III and the *Nhe*I sites in pCAL838 (*katA*), or the NheI and the SphI sites of pCAL838 (*mfd*, uvrB) (63) to form pHM724, pHM630, and pHM742. Plasmids were linearized with *Kpn*I and transformed into competent HM1, Δ*mfd* or Δ*uvrB* cells respectively. Cells were plated on MLS-containing plates and after overnight incubation at 37 °C, MLS-resistant colonies were tested for growth in media lacking threonine. Colonies that lack growth in media without threonine and were MLS resistant were selected as double crossover integrants.

For deleting the SID (38) of the endogenous *polA* gene in *B. subtilis*, 1 kb of homology on each side of the SID was amplified and cloned using NEBuilder® HiFi DNA Assembly Master Mix into the BamHI site of pMiniMAD2 (64), generating pHM736. This plasmid was transformed into RecA+ *E. coli* cells, purified, and transformed into HM1 *B. subtilis* cells, which were plated into MLS plates. After 48 h at room temperature, a single colony was streaked in a fresh MLS plate and grown at 42 °C for 8 h to force plasmid integration. A single colony was inoculated into liquid media and grown at 24 °C for 8 h, then diluted 1:30 and grown at 24 °C for 16 h; this was repeated for 3 d. Cells were then streaked on plates without antibiotics and grown at 37 °C overnight. Single colonies were then grown in plates with and without MLS at 42 °C for 8 h. For colonies that are MLS sensitive, DNA was extracted and a PCR surrounding the SID was performed and ran on native PAGE gel. A colony with an apparent deletion was sequenced to confirm the expected 30 bp deletion.

The *S. enterica* Typhimurium strains ST19 and SL1344 (22, 65) were a gift from Sam Miller (University of Washington) and Mariana Byndloss (Vanderbilt University) respectively, the *P. aeruginosa* strain is CF127 (66) and was a gift from Matt Parsek (University of Washington) and the multidrug-resistant *S. aureus* strain is a cystic fibrosis patient derived strain obtained from the Vanderbilt University Medical Center.

For *S. enterica*, knockouts were made from the SL1344 strain by recombineering as previously described (67) using the pSIM27 plasmid, a gift from the Court lab (https://redrecombineering.ncifcrf.gov/strains--plasmids.html). In short, for knocking out *mfd*, the chloramphenicol resistance gene was amplified from the pKD3 plasmid [a gift from the Wanner lab (68)] while adding 40 nucleotides of homology upstream of the start site and downstream of the stop codon using Q5 polymerase (*SI Appendix*, Table S2). The PCR amplicon was cleaned and electroporated into competent, wildtype cells harboring the pSIM27 plasmid. Chloramphenicol-resistant colonies were selected and checked by PCR (*SI Appendix*, Table S2). For knocking out *uvrB,* the kanamycin resistance gene was amplified from an *E. coli* strain with this gene on its chromosome (*SI Appendix*, Table S2) and recombineering was performed as described above.

All plasmids used in this study have been sequenced in full at Plasmidsaurus.

### MIC Determination.

To determine the MIC_50_ for all strains and antibiotics use, a single colony was inoculated and grown to an OD of 0.5 to 1 and diluted to an OD of 0.01 in culture media containing the indicated concentration of the antibiotic. Cells were grown at 37 °C for 16 h and the OD of each culture was measured.

### RNA Extraction and Gene Expression Levels Determination.

To measure the expression of the *katA* gene, single colonies were grown for 3 h in 10 mL of LB to reach exponential phase. Cultures were then diluted an OD of 0.05 in 10 mL of LB including 1 mM IPTG and cultured for an hour (three generations). RNA was extracted with the GeneJET RNA Purification Kit (Thermo) following the manufacturer’s instructions. 500 ng of RNA was treated with DNase I, RNase-free (Thermo), and cDNA was synthesized with the iScript™ cDNA Synthesis Kit (Biorad) using random primers in 20 µL reactions. Gene expression was determined by qPCR using the SsoAdvanced™ Universal SYBR® Green Supermix (Biorad) in 12 µL reactions, 5 µL of cDNA was used for the detection of *katA* and 2 µL for the detection of the 16S rRNA cDNA (used as housekeeping gene). Relative gene expression was calculated by the ΔΔCt method. For statistical comparison, ΔCt values were used.

### ROS Detection by Flow Cytometry.

ROS were detected with Dihydrorhodamine 123 (DHR) (19). 0.05 OD units of *S. enterica* ST19 on exponential phase were incubated with 250 µM of DHR in PBS for 30 min at 37 °C. Cells were then diluted 50-fold in PBS and ROS^+^ cells were detected by flow cytometry using a 3-Laser Fortessa. 100,000 cells per data point were measured. Gates for ROS^+^ cells were placed so that 0% of DMSO-only cells were positive. When indicated, 4 µg/mL rifampicin was added. A sublethal amount of kanamycin (5 µg/mL) was used as a positive control (17).

### Catalase Assay.

Catalase activity assay was performed as in ref. 69. 1 OD unit of exponential phase *B. subtilis* or stationary phase *S. enterica* was spun down and resuspended in 100 µL of PBS. The resuspended cells were then gently mixed with 100 µL of 1% Triton X-100 and 100 µL of 30% hydrogen peroxide. Samples were incubated for 15 min at RT. Using this assay, the amount of oxygen bubbles is directly proportional to the catalase activity (69)

### Evolution Assays.

Evolution assays were performed as previously described (15). A single colony of the indicated species and genotype was grown until an OD of 1 to 2 was reached. Culture was then diluted to an OD of 0.01 in culture media and grown in seven different concentrations of the indicated antibiotic, ranging from no antibiotic to 16× the MIC, as well as thiourea or IPTG when indicated. Cells were grown for 24 h at 37 °C with aeration, after which the OD was measured. The culture with the highest antibiotic concentration that showed an OD larger than 0.5× the OD of the culture without antibiotic (or, in the case of *P. aeruginosa*, an OD > 0.3) was diluted 100× (10× when the assay was performed in anaerobiosis) to an OD of approximately 0.01 and again grown in seven different antibiotic concentrations. This process was repeated six times unless the fastest evolving strains reach an MIC higher than the solubility of the antibiotic in media. When indicated, experiments were performed in an anaerobic chamber.

### Determination of the Mutation Rates by Fluctuation Assays.

Mutations rates were calculated as previously described (15). A single colony was inoculated into 2 mL of LB and grown for 2 h (*B. subtilis*) or 2.5 h (*S. enterica*) to reach exponential growth (0.1 < OD < 0.6). This culture was diluted to an OD of 0.0005 and between 3 and 10 parallel cultures with 2 mL of LB were grown for 4.5 h. Then, 1.5 mL of cells were pelleted and plated on 50 μg/mL rifampicin-containing plates. The remaining cells were serially diluted in 1× Spizizen media and plated on antibiotic-free media to quantify total viable cells. Colonies were counted after 24 h at 37 °C (rifampicin plates) or 16 h at 30 °C (no antibiotic plates). Mutation rates were calculated by using the Fluctuation AnaLysis CalculatOR (70), utilizing the Ma-Sandri-Sarkar maximum likelihood method.

### Growth Curves.

Growth curves were determined by growing a single colony of the indicated species until and OD of 1 to 2 was reached. The culture was diluted to an OD of 0.01 in culture media and growth in an Epoch microplate spectrophotometer (BioTek) at 37 °C for 16 h. OD600 was measured every 10 min.

### PolA Purification.

The coding sequence of PolA without the start codon was amplified by PCR using Q5 polymerase (NEB) and cloned BamHI-XhoI into pET28a (Thermo) to generate an N-terminal 6× his tagged protein coding sequence. The plasmid was transformed into BL21(DE) cells (NEB), and a single colony was inoculated into 70 mL of LB and grown overnight in LB containing kanamycin. Ten milliliters of culture was then inoculated in 1 L of LB+kanamycin and grown until an OD600 of 0.6, when 1 mM IPTG was added to the media. Cells were grown for 4 h and centrifuged for 15 min at 4,000g. Pellets were resuspended in 30 mL of CelLytic B cell lysis reagent (Sigma) with 3 µL of Benzonase (Sigma) and 10 mM imidazole and shaken at RT for 10 min. Lysate was centrifuged at 20,000g at 4 °C and the supernatant was mixed with an equal volume of equilibration buffer (20 mM sodium phosphate pH 7.4, 300 mM sodium chloride, 10 mM imidazole), and run twice through 15 mL of equilibrated HisPur™ Ni-NTA Resin (Thermo) at 4 °C. Resin was washed with 150 mL of wash buffer (20 mM sodium phosphate pH 7.4, 300 mM sodium chloride, 40 mM imidazole) and eluted with 15 mL of elution buffer (20 mM sodium phosphate pH 7.4, 300 mM sodium chloride, 150 mM imidazole). Protein was dialyzed with a 30-mL Slide-A-Lyzer Dialysis Cassette G2 20000 MWCO (Thermo) against 10 mM tris pH 8, 50 mM NaCl, 5% glycerol, 0.1 mM DTT, 0.1 mM EDTA overnight at 4 °C Protein prep was then concentrated with Amicon Ultra-15 Centrifugal Filter Units 3000K (Millipore) to a final concentration of 1.6 mg/mL measured by Bradford assay (Thermo).

For the preparation of PolA-ΔSID, pET28a-PolA was used as template for PCR with 5′ phosphate-containing primers, separated by the SIM sequence (amino acids 469 to 478) going outwards using Q5 polymerase (NEB). PCR product was purified, digested with DpnI (NEB) to eliminate the template plasmid, purified, and ligated using T4 DNA ligase for 1 h at room temperature. The purification was performed as described above for the wild-type protein to a concentration of 2.1 mg/mL. Both protein preps were run on a 10% SDS-PAGE and stained by Imperial protein stain (Thermo) to confirm purity of enzyme.

### PolA Synthesis Assay.

PolA synthesis was tested on 40 mM Tris pH 8, 10 mM MgCl_2_, 60 mM KCl, 2.5% glycerol buffer containing 1 mM dNTPs, 1.5 nM of the indicated DNA substrate labeled with Cy5, and 100 nM PolA. Ten microliter reactions were incubated at 37 °C for 10 min and stopped with 10 μL of 95% formamide 10 mM EDTA. DNA was denatured at 85 °C for 15 min and run in a 12% urea denaturing gel at 150 V for 30 min. Gel was scanned in a ChemiDoc imaging system (BioRad).

The substrates for PolA synthesis experiments were done by annealing three (gap substrate) or two (primer extension substrate) HPLC purified oligos (Sigma) in a thermocycler. The template for the abasic site substrate contained a deoxyuracil in the ninth position. The abasic site was generated by treating the annealed oligo with hSMUG1 (NEB) for 30 min at 37 °C followed by heat inactivation of the enzyme at 65 °C for 20 min.

## Supplementary Material

Appendix 01 (PDF)

## Data Availability

All study data are included in the article and/or *SI Appendix*. All materials generated will be shared upon request.
