# Supplementary material for: Oxidative stress drives mutagenesis through transcription-coupled repair in bacteria
Source: Proc Natl Acad Sci U S A. 2023 Jun 26;120(27):e2300761120. doi: 10.1073/pnas.2300761120 (PMC10318952; doi:10.1073/pnas.2300761120)
Supplement: Supplementary file 1 — Appendix 01 (PDF) [file pnas.2300761120.sapp.pdf]

PNAS

Supporting Information for
Oxidative stress drives mutagenesis through transcription-coupled repair in bacteria

Juan Carvajal-Garcia, Ariana N. Samadpour, Angel J. Hernandez Viera, Houra Merrikh

Houra Merrikh
Email: houra.merrikh@vancerbilt.edu

This PDF file includes:

Figures S1 to S3
Tables S1 to S2

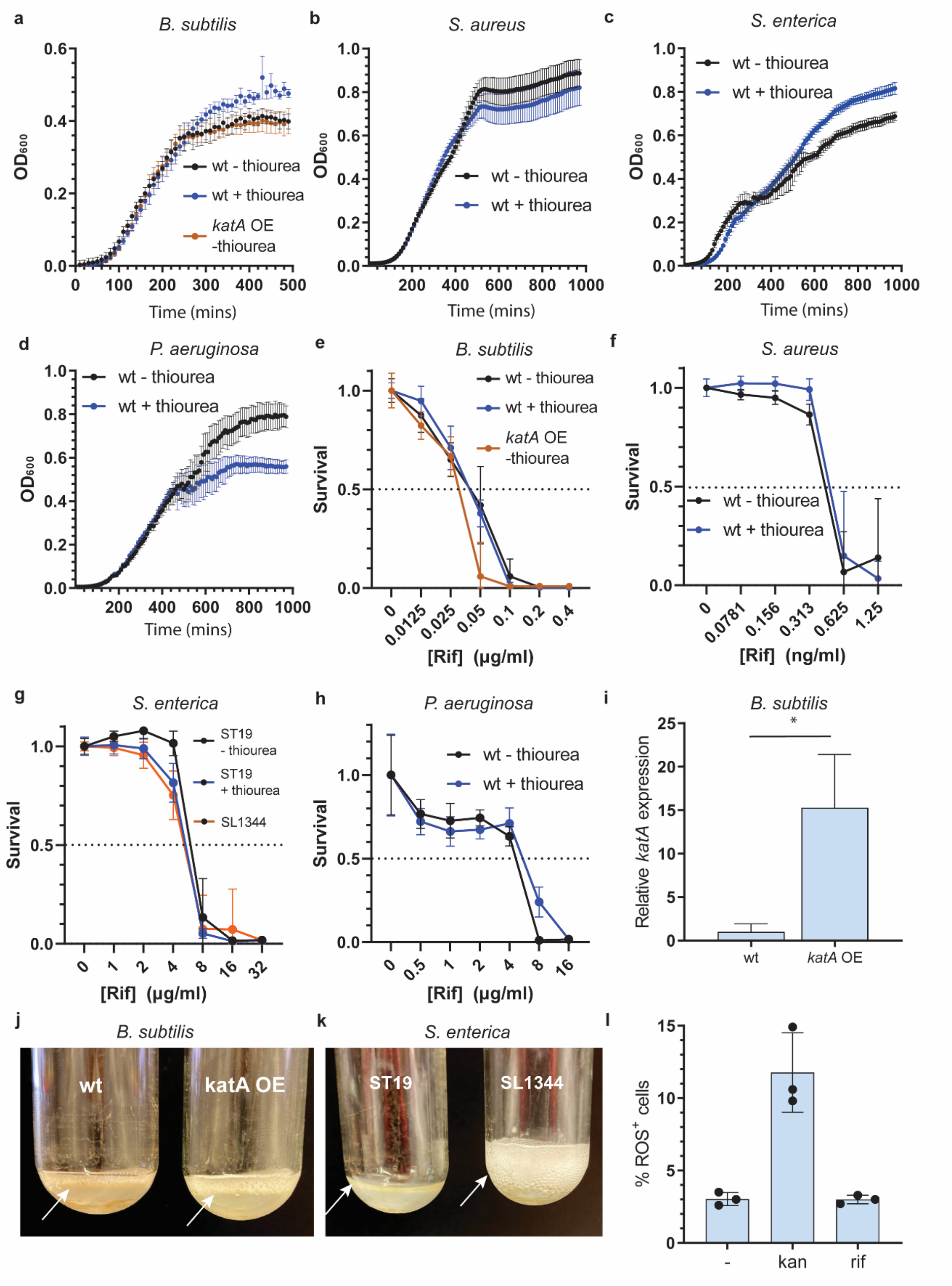

Fig. S1. a-c) OD600 measured every 10 mins for the indicated time in a cultures of *B. subtilis* (a), *S. aureus* (b), and *S. enterica* serovar Typhimurium ST19 (c), with and without 50 mM thiourea in the media. 1 mM IPTG was added to the *katA* OE strain. n=12 biological replicates. Error bars represent the standard deviation d) OD600 measured every 10 mins for the indicated time in a

culture of *P. aeruginosa* with and without 10 mM thiourea in the media, n=12 biological replicates. Error bars represent the standard deviation e-h) Survival of the indicated strain to the indicated concentration of rifampicin after 16 hours of growth at 37 °C. The same concentrations of thiourea as in panels a-d were added when indicated. 1 mM IPTG was added to the *katA* OE strain. n=12 biological replicates. Error bars represent the standard deviation i) Normalized katA cDNA detected by qPCR in wild-type and katA overexpressing cells in the presence of 1 mM IPTG, n=5 biological replicates. Statistical significance was assessed by a two-tailed t-test, *$p<0.05$ j-k) Cells of the indicated species were mixed with equal volumes of 1% Triton X-100 and 30% H_2O_2 to assay global catalase activity. The formation of oxygen bubbles (arrows) is directly proportional to the amount of catalase activity l) Percent *S. enterica* cells that are ROS^+ as determined by flow using the dye DHR123 in the absence of antibiotic, 5 µg/ml of kanamycin or 4 µg/ml rifampicin

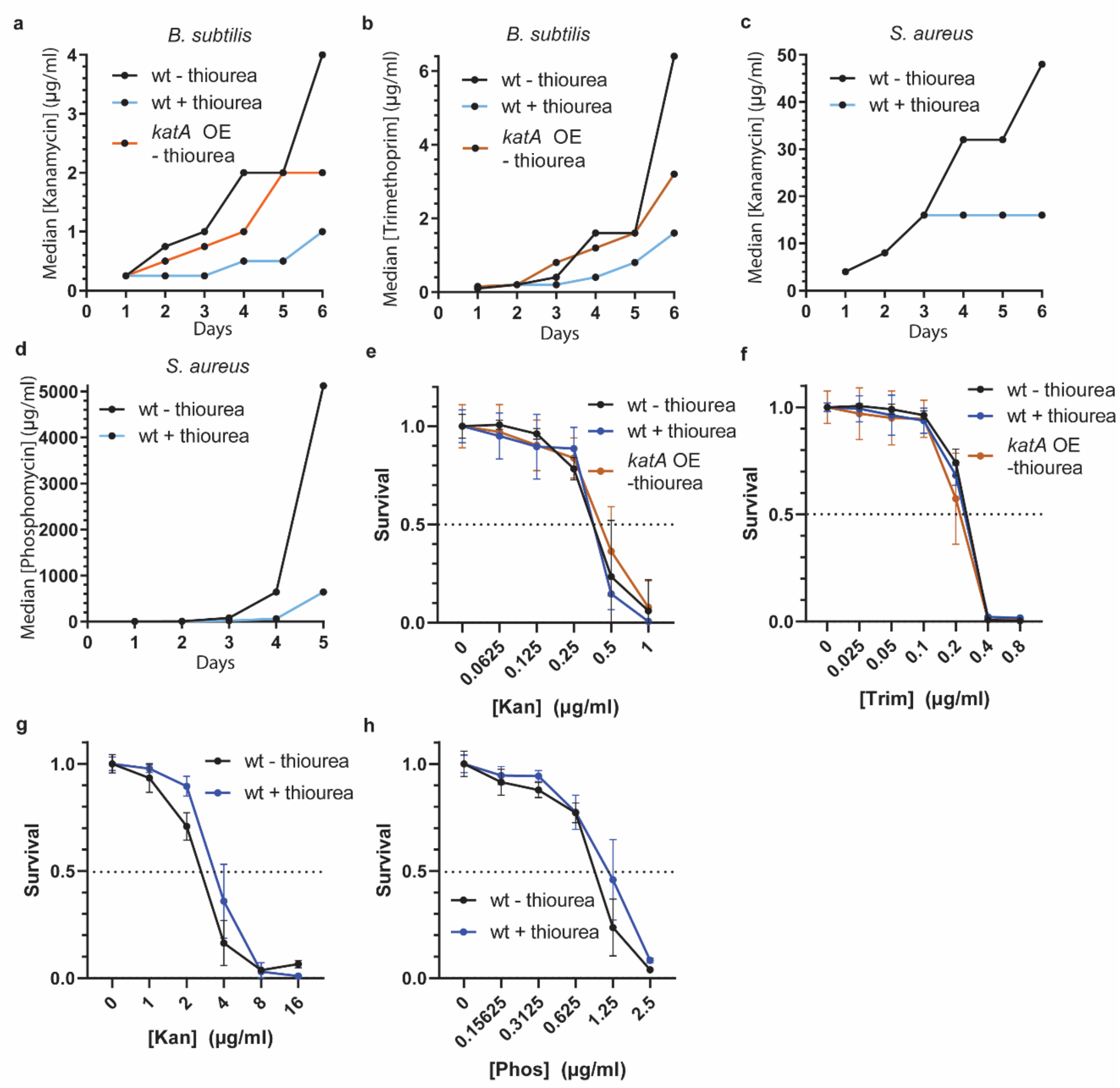

Fig. S2. a-d) Median concentration of antibiotic that allows for growth in the indicated strains at each sampled timepoint. 50 mM thiourea was included in the media where indicated. 1mM IPTG was added for *katA* overexpression. n=24 (wt – thiourea, kanamycin), 11 (wt + thiourea, kanamycin), 12 (*katA* overexpression, kanamycin), 12 (wt – thiourea, trimethoprim), 12 (wt + thiourea, trimethoprim), 12 (*katA* overexpression, trimethoprim), and 12 for all *S. aureus* experiments. e-h) Survival of the indicated species to the indicated concentration of the indicated antibiotics after 16 hours of growth at 37 °C. 50 mM thiourea was added when indicated. 1 mM IPTG was added to the *katA* OE strain. n=12 biological replicates. Error bars represent the standard deviation

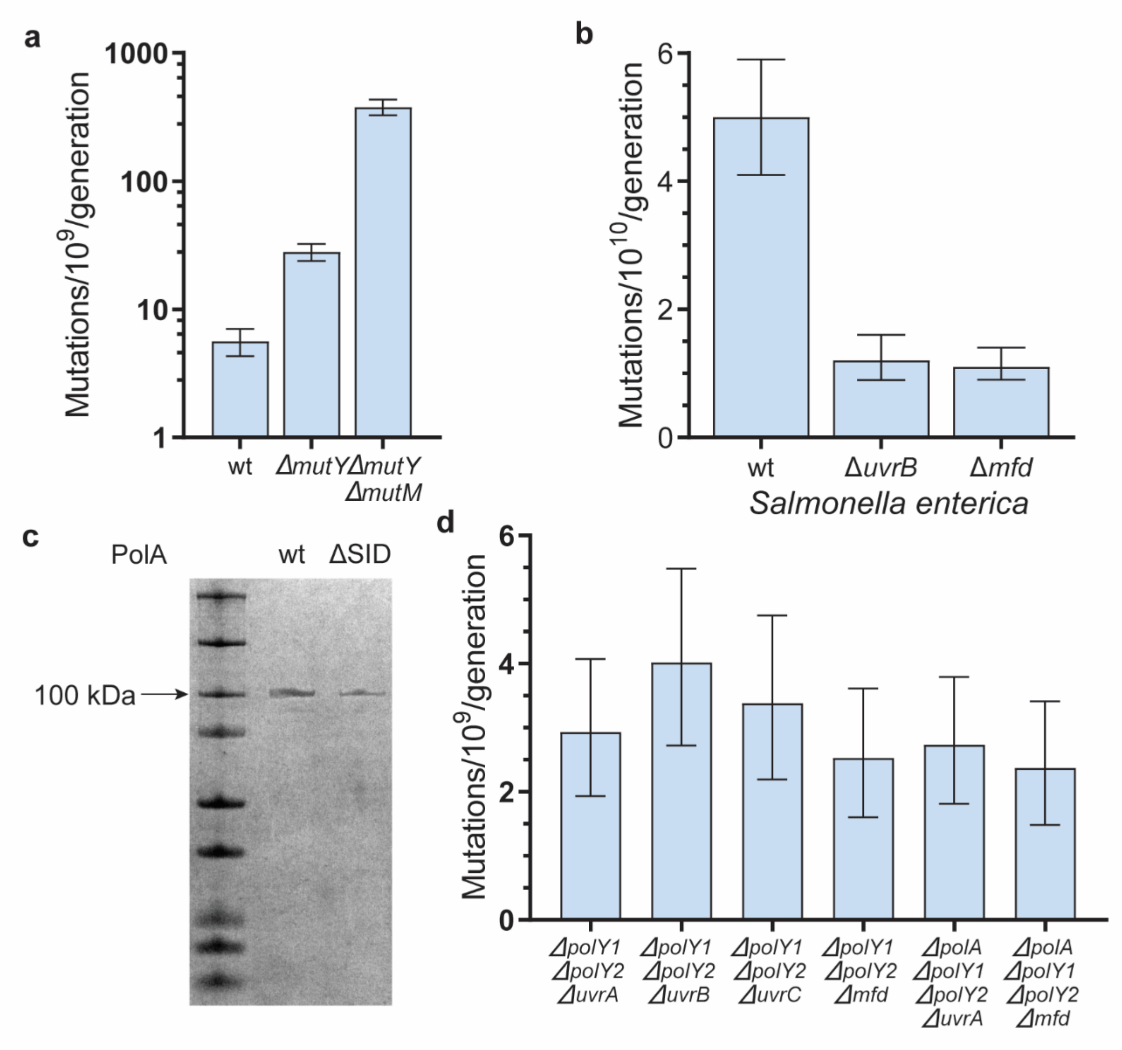

Fig. S3. a) Mutation rates of *Bacillus subtilis* strains of the indicated genotype to rifampicin, n=51 (wt), 59 (Δ*mutY*), 21 (Δ*mutY*, Δ*mutM*) b) Mutation rates of *S. enterica* serovar Typhimurium strain ST19 measured using rifampicin. n=54 (wt), 40 (ΔuvrB), 48 (Δmfd). c) SDS-PAGE of purified *B. subtilis* PolA and PolA-ΔSID. d) Mutation rates of *Bacillus subtilis* strains of the indicated genotype to rifampicin. n=40 (Δ*polY1* Δ*polY2* Δ*uvrA*), 40 (Δ*polY1* Δ*polY2* Δ*uvrB*), 30 (Δ*polY1* Δ*polY2* Δ*uvrC*), 33 (Δ*polY1* Δ*polY2* Δ*mfd*), 36 (Δ*polA* Δ*polY1* Δ*polY2* Δ*uvrA*), 36 (Δ*polA* Δ*polY1* Δ*polY2* Δ*mfd*) biological replicates. Error bars are 95% confidence intervals.

Table S1. Strains used.

Strain	Species	Genotype	Reference	Figure
HM1	*B. subtilis*	wt	Brehm 1973	1, 2, 3, 4, S1, S2, S3
HM2521	*B. subtilis*	*mfd::MLS*	Million-Weaver 2015	2, 3
HM2633	*B. subtilis*	*uvrA::MLS*	This study	2, 3
HM2634	*B. subtilis*	*uvrB::MLS*	This study	2
HM2635	*B. subtilis*	*uvrC::MLS*	This study	2
HM2472	*B. subtilis*	*mfd::markerless uvrA::MLS*	This study	2
HM2473	*B. subtilis*	*mfd::markerless uvrB::MLS*	This study	2
HM2474	*B. subtilis*	*mfd::markerless uvrC::MLS*	This study	2
HM4640	*B. subtilis*	*mfd::kan thrC::Pspank(hy) mfd*	This study	2
HM4648	*B. subtilis*	*uvrB::kan thrC::Pspank(hy) uvrB*	This study	2
HM3533	*B. subtilis*	*polA::MLS*	This study	4
HM4449	*B. subtilis*	*uvrA::markerless polA::MLS*	This study	4
HM3550	*B. subtilis*	*mfd::markerless polA::MLS*	This study	4
HM391	*B. subtilis*	*polY1::Cm*	Million-Weaver 2015	4
HM345	*B. subtilis*	*polY2::Cm*	Million-Weaver 2015	4
HM2632	*B. subtilis*	*polY1::MLS polY2::Cm*	This study	4
HM3567	*B. subtilis*	*polY1::markerless polY2::Cm polA::MLS*	This study	4
HM3116	*B. subtilis*	*mutY::MLS*	This study	S3
HM3123	*B. subtilis*	*mutY::markerless mutM::MLS*	This study	S3
HM2666	*B. subtilis*	*polY1::markerless polY2::Cm uvrA::MLS*	This study	S3
HM2667	*B. subtilis*	*polY1::markerless polY2::Cm uvrB::MLS*	This study	S3
HM2668	*B. subtilis*	*polY1::markerless polY2::Cm uvrC::MLS*	This study	S3
HM2669	*B. subtilis*	*polY1::markerless polY2::Cm mfd::MLS*	This study	S3
HM4488	*B. subtilis*	*polY1::markerless polY2::Cm mfd::markerless polA::MLS*	This study	S3
HM4482	*B. subtilis*	*polY1::markerless polY2::Cm*	This study	S3

		uvrA::markerless polA::MLS		
HM4502	*B. subtilis*	*thrC::Pspank(hy) katA*	This study	1, S1, S2
HM2212	*P. aeruginosa*	*CF127*	Wolfgang 2003	1, S1
HM4318	*S. aureus*	penicillin, oxacillin, erythromycin resistant	This study	1, S1, S2
HM1996	*S. enterica ST19*		Hayden et al., 2016	1, S1
HM4315	*S. enterica SL1344*		Hoiseth and Stocker 1981	1, 2, S1, S3
HM4500	*S. enterica*	*mfd::Cm*	This study	S3
HM4510	*S. enterica*	*uvrB::Kan*	This study	S3
HM4554	*B. subtilis*	*PolA-ΔSID*	This study	4

Table S2. Oligonucleotides used

PCR/substrate	Species	Oligo	Sequence (5'->3')
uvrA::markerless	*B. subtilis*	Fwd	GGAGCTTCGCGATTTACTTTTAG
		Rev	GCTTGCCTGCTAAGCCC
mfd::markerless	*B. subtilis*	Fwd	CGAAATCCGCATTACCACGA
		Rev	TTAGGAATCACGACCCGACC
polY1::markerless	*B. subtilis*	Fwd	TGTTACGGCGCTGTGTATC
		Rev	CGAATTCATGCGGAAGACTTTAC
mutY::markerless	*B. subtilis*	Fwd	TCGTACTGTGCCCTTAGTGT
		Rev	TGGAAGAACAGTGAACTCGC
uvrB recombineering	*S. enterica*	Fwd	TACACCCCTGCCCGCTCACTCCTTCAGGTAGCCGCTCATGTATGGACAGCAAGCGAACCG
		Rev	CCATGGTAACGATGACTCGCTGGCGATCGACACATTGTCATCAGAAGAACTCGTCAAGAAG
mfd recombineering	*S. enterica*	Fwd	GACGCCCGGCCTGACGCTTATGCAATAGCGTTTTCTTCCAGTGTAGGCTGGAGCTGCTTC
		Rev	GTGCGGCGTAAAACAAAAAGAGATACTGACAACCGTTATGCATATGAATATCCTCCTTAG
uvrB check	*S. enterica*	Fwd	GCAATATTCACCGTCGAGAG
		Rev	CTATTGCACTGAAATTCTCAAAGC
mfd check	*S. enterica*	Fwd	AGAATTTGTAAAGATTAGGCCGG
		Rev	TGAAGCAGCCTGAAGGG
Gap substrate	*In vitro*	Top left	Cy5-GCCTAGCTCTGCCATGCATA
		Top right	TACACCTGTCTATCATTAGT
		Bottom	ACTAATGATAGACAGGTGTAGTACGGAAATCTTCTACGTTTATGCATGGCAGAGCTAGGC
Primer extension substrate, template without abasic site	*In vitro*	Top	Cy5-ATTCTGGTGGAAATGGCGCGCTGCTAT
Primer extension substrate, template with abasic site	*In vitro*	Bottom	GTGGAACGCTATATGTGCCATATAGCAGCGCGCCATTTCCACCAGAAT
Abasic site substrate	*In vitro*	Top	Cy5-ATTCTGGTGGAAATGGCGCGCTGCTAT
		Bottom	GTGGAACGCTA[dU]ATGTGCCATATAGCAGCGCGCCATTTCCACCAGAAT
polA for cloning into pET28a	*B. subtilis*	Fwd	AAGGATCCACGGAACGAAAAAAATTAGTGCTTGTAGAC

		Rev	AAGAATTCTTATTTCGCATCGTACCAAGATGGGC
katA for cloning into pCAL838	*B. subtilis*	Fwd	TTAAGCTTATGAGTTCAAATAAACTGACAACTAGCTGGG
		Rev	TTGCTAGCTTAAGAATCTTTTTAATCGGCAATCCAAGGC
uvrB for cloning into pCAL838	*B. subtilis*	Fwd	TTGCTAGCCGGACATAATGAATATAAAGACTG
		Rev	TTGCATGCTTGTTCATCATCCTTCCG
katA qPCR	*B. subtilis*	Fwd	GAGTCACCTGAGGATAAGCAAG
		Rev	GGCTTGAGTGTAGTGATCGTAG
16S RNA qPCR	*B. subtilis*	Fwd	GACATCCTCTGACAATCCTAGAG
		Rev	GGCAGTCACCTTAGAGTGCCCAAC
polA for cloning into pET28a	*B. subtilis*	Fwd	AAGGATCCACGGAACGAAAAAAATTAGTGCTTGTAGAC
		Rev	AAGAATTCTTATTTCGCATCGTACCAAGATGGGC
polA$^{\Delta SID}$ for cloning into pET28a	*B. subtilis*	Fwd	5’ Phos-CTCTTGAACGAGCTTTTCCCGAAG
		Rev	5’ Phos-GAAGAGCTGGAAATGCCTCTTGC
Left *polA*$^{\Delta SID}$ homology arm for cloning into pMiniMAD2	*B. subtilis*	Fwd	CCTGCAGGTCGACTCTAGAGAACGACAGTTGCCATTACGAGAAAG
		Rev	AGAGGCATTTCCAGCTCTTCCTCTTGAACGAGCTTTTCCCG
Right *polA*$^{\Delta SID}$ homology arm for cloning into pMiniMAD2	*B. subtilis*	Fwd	GGGAAAAGCTCGTTCAAGAGGAAGAGCTGGAAATGCCTCTTG
		Rev	GAGCTCGGTACCCGGGGATCTTATTTCGCATCGTACCAAGATGGG
polA$^{\Delta SID}$ check	*B. subtilis*	Fwd	AGGAGCAAAACGGGCAGTGC
		Rev	ACGCCAGTTGATTCCATTTCGC
